# Comparison of Complete Versus Incomplete Percutaneous Revascularization in Patients With Chronic Total Occlusion: A Systematic Review and Meta-Analysis

**DOI:** 10.7759/cureus.66759

**Published:** 2024-08-13

**Authors:** Tanya Sinha, Bhanu Chaudhary, Yoseph L Herpo, Naiha Talha, Fareed Baksh, Muhammad Arsalan, Faria Khilji, Shamsha Hirani

**Affiliations:** 1 Internal Medicine, Tribhuvan University, Kathmandu , NPL; 2 Surgery, Southern Illinois University School of Medicine, Carbondale , USA; 3 Internal Medicine, Hayat medical college, Addis Ababa, ETH; 4 Internal Medicine, Allama Iqbal Medical College, Lahore, PAK; 5 Medicine, Allama Iqbal Medical College, Lahore, PAK; 6 Internal Medicine, Medical Teaching Institute, Lady Reading Hospital, Peshawar, PAK; 7 Internal Medicine, Tehsil Headquarter Hospital, Shakargarh, PAK; 8 Internal Medicine, Quaid-e-Azam Medical College, Bahawalpur, PAK; 9 Cardiology, Baqai Hospital, Karachi, PAK

**Keywords:** systematic review and meta analysis, chronic total occlusion, incomplete, complete, percutaneous revascularization

## Abstract

The optimal extent of revascularization in patients with chronic total occlusion (CTO) undergoing percutaneous coronary intervention (PCI) remains debated. This meta-analysis aimed to compare the clinical outcomes of complete versus incomplete revascularization in CTO patients. A systematic search of EMBASE, PubMed, and Web of Science was conducted up to July 6, 2024. Studies reporting outcomes in CTO patients undergoing PCI with complete or incomplete revascularization were included. The primary outcomes were major adverse cardiovascular events (MACE), all-cause mortality, and cardiovascular mortality. Eight studies with a total of 7,067 patients (4,854 complete and 2,213 incomplete revascularization) were included. Complete revascularization was associated with a significantly lower risk of MACE (RR: 0.57, 95% CI: 0.43-0.77), all-cause mortality (RR: 0.54, 95% CI: 0.37-0.78), and cardiovascular mortality (RR: 0.46, 95% CI: 0.29-0.75) compared to incomplete revascularization. There was no significant difference in the risk of recurrent myocardial infarction between the two groups (RR: 0.60, 95% CI: 0.20-1.80). In patients with CTO undergoing PCI, complete revascularization is associated with significantly better clinical outcomes, including lower risks of MACE, all-cause mortality, and cardiovascular mortality, compared to incomplete revascularization. These findings suggest that achieving complete revascularization should be prioritized when feasible in CTO patients.

## Introduction and background

Chronic total occlusion (CTO) is defined as an occlusion of an epicardial coronary artery without antegrade flow through the lesion and with a probable or definite duration of ≥3 months [1]. These lesions are characterized by heavy calcification and fibrosis, making them challenging to treat with conventional percutaneous coronary intervention (PCI) techniques. CTOs are found in approximately 15-30% of patients undergoing coronary angiography and are associated with increased morbidity and mortality if left untreated [2-3]. 

Management of CTOs has evolved significantly over the past decades. While coronary artery bypass grafting (CABG) was once the primary treatment option, advances in interventional techniques and technologies have made percutaneous revascularization an increasingly viable alternative [4]. PCI for CTOs, however, remains one of the most technically demanding procedures in interventional cardiology, requiring specialized skills, equipment, and experience [5]. Percutaneous revascularization of CTOs aims to restore blood flow to the occluded vessel, potentially improving symptoms, left ventricular function, and long-term outcomes [6]. However, the optimal extent of revascularization in CTO patients remains a subject of debate. Complete revascularization involves treating all significant coronary lesions, including the CTO, while incomplete revascularization may leave one or more lesions untreated, often due to technical difficulties or perceived risk-benefit considerations. Coronary lesions are areas of damage or abnormality in the coronary arteries, often caused by atherosclerosis, leading to narrowed or blocked arteries [7]. 

The comparison between complete and incomplete revascularization in CTO patients is of particular interest, as it may have significant implications for patient outcomes and resource utilization. While complete revascularization theoretically offers the potential for superior myocardial perfusion and functional improvement, it may also be associated with longer procedure times, increased radiation exposure, and higher complication rates. Conversely, incomplete revascularization might provide symptomatic relief with potentially lower procedural risks but may not fully address the underlying coronary disease burden [8-9]. 

The clinical significance of complete revascularization in patients with chronic total occlusions (CTO) is still not well understood. Additionally, there is an absence of recommendations from societal guidelines regarding the pursuit of complete revascularization. The 2019 European Society of Cardiology guideline document [10] recently underscored this current evidence gap. This uncertainty is primarily due to the use of non-standardized definitions for complete and incomplete revascularization (ICR) in earlier studies, a shortage of randomized clinical data, differences in revascularization techniques, and heterogeneous study populations. Given the ongoing controversy surrounding this topic and the lack of clear consensus in current guidelines, a comprehensive meta-analysis of existing studies comparing complete versus incomplete percutaneous revascularization in CTO patients is warranted. The aim of this meta-analysis is to systematically evaluate the available evidence on the efficacy and safety of these two approaches, focusing on clinically relevant outcomes such as mortality, major adverse cardiac events (MACE), cardiovascular mortality, myocardial infarction, and repeated revascularization. 

## Review

Methodology 

The Preferred Reporting Items for Systematic reviews and Meta-Analyses (PRISMA) guidelines were followed. 

Search Strategy 

A search was conducted from the databases' creation date until July 6, 2024, using EMBASE, PubMed, and Web of Science. The following search phrases were employed: "Percutaneous coronary intervention" (PCI), "Incomplete revascularization" (OR "Complete revascularization"), and "Chronic total occlusion" (CTO). When possible, these keywords were also looked up in exploding medical subject titles in addition to text terms. All language studies were included. We looked for other pertinent publications in the bibliographies of the included research and pertinent review articles. Two writers conducted the search independently, and any discrepancy between them was settled by consensus. 

Study Selection 

Based on the research design or the PCI indication, studies of patients with CTO who underwent PCI and reported mortality or cardiovascular events among patients with and without complete revascularization were chosen. Excluded from consideration were publications that did not report desired outcomes. Excluded from the studies were patients who weren't CTOs. Reviews, editorials, case series, and case reports were also disregarded. Every title and abstract was independently reviewed by two reviewers to look for research that would fit the inclusion requirements. After obtaining the complete reports of these investigations, data on the study design, participant characteristics, comprehensive revascularization definition, outcome events, and follow-up were extracted separately. 

Outcomes 

Outcomes assessed in this meta-analysis included MACE, all-cause mortality, cardiovascular mortality, recurrent myocardial infarction, and repeated revascularization. MACE refers to Major Adverse Cardiovascular Events, including heart attack, stroke, cardiovascular death, and revascularization. Data related to study outcomes were extracted by two authors independently and any disagreement between authors was resolved through discussion. 

*Statistical Analysis* 

A random-effects meta-analysis was performed using RevMan Version 5.4.1 (Cochrane, London, United Kingdom). The 95% confidence interval (CI) and risk ratio (RR) were computed. When available, propensity-matched or adjusted risk estimates were applied. The consistency between studies was evaluated using the Cochrane Q-statistic (I2), where I2 <25% was deemed low heterogeneity, I2 >50% moderate, and I2 >75% high. 

Results 

**Figure 1:**
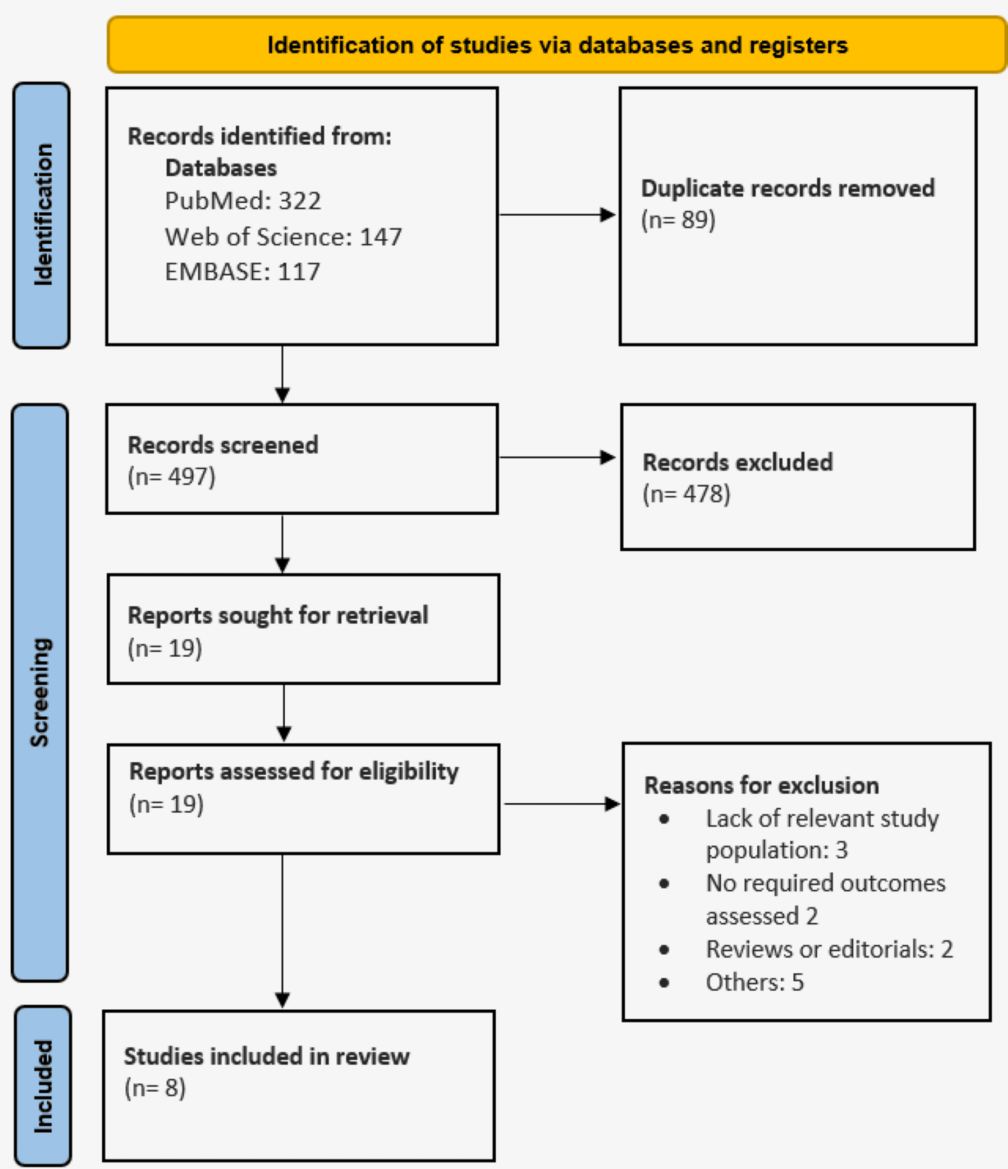
PRISMA flowchart of study selection process PRISMA: Preferred Reporting Items for Systematic reviews and Meta-Analyses

The electronic database search yielded 586 studies. Following duplicate removal, 497 studies were initially screened using titles and abstracted, followed by a full-text screening of 19 studies. Finally, eight studies were included in this meta-analysis. Figure 1 shows the PRISMA flowchart of study selection. Table 1 shows the characteristics of the studies included. Out of eight included studies, three were conducted in Italy, two in China, and one each in the United States, India, and Spain. The pooled sample size is 7067 (4854 in complete vascularization and 2213 in incomplete vascularization). 

**Table 1:** Included studies' characteristics

Author Name	Year	Study Design	Study Region	Complete	Incomplete	Follow-up
Danzi et al [11]	2013	Retrospective	Italy	76	44	12 Months
Goel et al [12]	2017	Retrospective	India	410	139	2.9 Years
Hannan et al [13]	2009	Retrospective	United States	3499	1233	18 Months
Li et al [14]	2023	Prosppective	China	190	122	21 Months
Maestre-luque et al [15]	2024	Retrospective	Spain	167	192	42 Months
Valenti et al [16]	2009	Retrospective	Italy	301	226	24 Months
Valenti et al [17]	2019	Retrospective	Italy	170	170	60 Months
Wu et al [18]	2022	Retrospective	China	41	87	38.02 Months

Meta-analysis of Outcomes 

**Figure 2:**
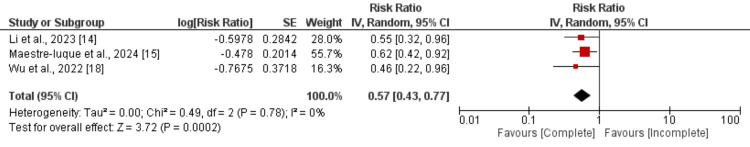
Comparison of MACE between complete and incomplete revascularization Sources: References [14-15, 18] MACE: major adverse cardiovascular events

MACE: Three studies were combined to evaluate the effect of complete vascularization on MACE among patients with CTO and the results of the meta-analysis are presented in Figure 2. Pooled analysis showed that the risk of MACE was significantly lower in patients with complete vascularization compared to incomplete vascularization (RR: 0.57, 95% CI: 0.43 to 0.77). No heterogeneity has been found among the study results (I-square: 0%). 

**Figure 3:**
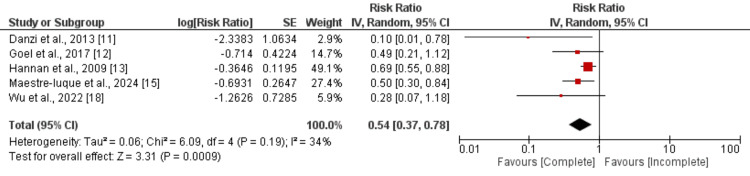
Comparison of all-cause mortality between complete and incomplete revascularization Sources: References [11-13, 15, 18]

All-cause Mortality: Five studies were combined to evaluate the effect of complete vascularization on all-cause mortality among patients with CTO, and the results of the meta-analysis are presented in Figure 3. Pooled analysis revealed that the risk of all-cause mortality was significantly lower in patients with complete vascularization compared to those with incomplete vascularization (RR: 0.54, 95% CI: 0.37 to 0.78). The study results showed low heterogeneity (I-square: 34%).

**Figure 4:**
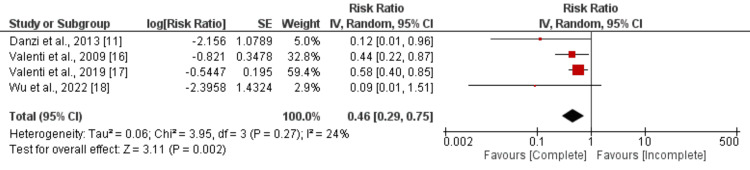
Comparison of cardiovascular mortality between complete and incomplete revascularization Sources: References [11, 16-18]

Cardiovascular Mortality: Four studies were combined to evaluate the effect of complete vascularization on cardiovascular mortality among patients with CTO, and the results of the meta-analysis are presented in Figure 4. Pooled analysis revealed that the risk of cardiovascular mortality was significantly lower in patients with complete vascularization compared to those with incomplete vascularization (RR: 0.46, 95% CI: 0.29 to 0.75). The study results showed low heterogeneity (I-square: 24%).

**Figure 5:**
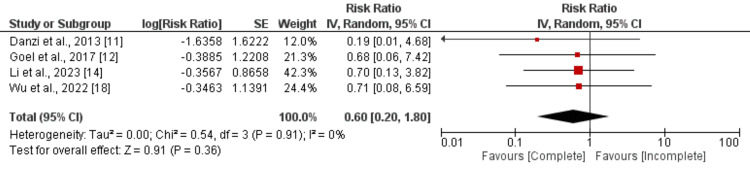
Comparison of recurrent myocardial infarction between complete and incomplete revascularization Sources: References [11-12, 14, 18]

Recurrent Myocardial Infarction: Four studies were combined to evaluate the effect of complete vascularization on recurrent myocardial infarction among patients with CTO, and the results of the meta-analysis are presented in Figure 5. Pooled analysis revealed that the risk of recurrent myocardial infarction was not different between complete vascularization and incomplete vascularization (RR: 0.60, 95% CI: 0.20 to 0.1.80). The study results showed no heterogeneity (I-square: 0%).

Discussion 

In our meta-analysis of eight studies, including over 7067 patients with CTO undergoing PCI, we found that the risk of MACE, all-cause mortality, and cardiovascular mortality was significantly lower in patients with complete vascularization compared to incomplete vascularization. To the best of our knowledge this is the first meta-analysis focusing on CTO as previous meta-analysis included patients with coronary artery disease. Lu et al. performed a meta-analysis that included acute coronary syndrome patients with multivessel disease undergoing PCI - complete vascularization demonstrated superior long-term outcomes compared to incomplete vascularization [19]. 

Our findings underscore the importance of achieving complete revascularization in patients with chronic total occlusion (CTO) undergoing percutaneous coronary intervention (PCI). The significant reduction in major adverse cardiovascular events (MACE), all-cause mortality, and cardiovascular mortality associated with complete revascularization suggests that this approach may offer substantial clinical benefits. These results align with the growing body of evidence supporting the advantages of complete revascularization in various subsets of coronary artery disease patients [20-21]. 

When comparing our results to previous studies, it's important to note the specificity of our focus on CTO patients. While Lu et al.'s [19] meta-analysis demonstrated superior long-term outcomes with complete revascularization in acute coronary syndrome patients with multivessel disease, our study extends these findings to the CTO population. This is particularly relevant given the unique challenges associated with CTO interventions, such as technical complexity and higher procedural risks [22-23]. 

Most of the studies in this analysis are based on registry data, meaning that the decision by the operator not to perform complete revascularization may reflect uncaptured comorbidities or the general frailty of the patient, acting as an indicator of poor health status, which contributes to the poorer reported outcomes. Despite nearly all studies adjusting for differences in baseline characteristics, the possibility of unmeasured confounding remains significant, particularly in registry-based studies. Additionally, the higher risk associated with incomplete revascularization may be related to the complexity or extent of coronary artery disease at baseline. 

This meta-analysis has several limitations. Firstly, our analysis cannot compare the outcomes of patients undergoing incomplete revascularization (IR) in different contexts, such as elective procedures versus acute coronary syndrome (ACS), because most studies do not report outcomes based on clinical presentation. Secondly, while we report an association between incomplete revascularization and adverse clinical outcomes, a causal relationship cannot be inferred. Although we have demonstrated this association, it should not be assumed that treating patients with additional PCI to achieve complete revascularization would necessarily improve their prognosis. Finally, only eight studies were included in this meta-analysis, and most of the outcomes were not assessed by the majority of the studies. We were also unable to perform meta-regression due to the limited number of studies that provided the baseline characteristics of the participants. 

Our findings have important implications for both clinical practice and future research. Clinically, the results strongly suggest that healthcare providers should prioritize complete revascularization in CTO patients undergoing PCI when feasible, as this approach may significantly improve long-term outcomes. This could influence treatment strategies, patient counseling, and resource allocation in cardiac care units. However, the observational nature of most included studies underscores the need for prospective, randomized controlled trials to definitively establish the causal relationship between complete revascularization and improved outcomes in CTO patients. Such trials should aim to address the limitations of current evidence, including potential confounding factors and the impact of baseline disease complexity. Additionally, future research should explore the optimal techniques and technologies for achieving complete revascularization in CTO cases, as well as investigate potential subgroups of patients who might benefit most from this approach. These efforts would help refine clinical guidelines and improve the management of this challenging patient population. 

## Conclusions

This meta-analysis of eight studies encompassing 7,067 CTO patients undergoing PCI demonstrates significant benefits of complete revascularization over incomplete revascularization. Complete revascularization was associated with lower risks of MACE, all-cause mortality, and cardiovascular mortality. These findings emphasize the importance of striving for complete revascularization in CTO patients when feasible. However, the observational nature of most included studies highlights the need for prospective, randomized trials to establish a causal relationship and address potential confounding factors. Future research should focus on optimizing techniques for complete revascularization in CTO cases and identifying patient subgroups who may benefit most from this approach. These efforts will help refine clinical guidelines and improve outcomes in this complex patient population.
